# Genetic responses to seasonal variation in altitudinal stress: whole-genome resequencing of great tit in eastern Himalayas

**DOI:** 10.1038/srep14256

**Published:** 2015-09-25

**Authors:** Yanhua Qu, Shilin Tian, Naijian Han, Hongwei Zhao, Bin Gao, Jun Fu, Yalin Cheng, Gang Song, Per G. P. Ericson, Yong E. Zhang, Dawei Wang, Qing Quan, Zhi Jiang, Ruiqiang Li, Fumin Lei

**Affiliations:** 1 Key Laboratory of Zoological Systematics and Evolution, Institute of Zoology, Chinese Academy of Sciences, Beijing 100101, China; 2 Novogene Bioinformatics Institute, Beijing 100083, China; 3 Department of Zoology, Swedish Museum of Natural History, PO Box 50007, SE-10405 Stockholm, Sweden

## Abstract

Species that undertake altitudinal migrations are exposed to a considerable seasonal variation in oxygen levels and temperature. How they cope with this was studied in a population of great tit (*Parus major*) that breeds at high elevations and winters at lower elevations in the eastern Himalayas. Comparison of population genomics of high altitudinal great tits and those living in lowlands revealed an accelerated genetic selection for carbohydrate energy metabolism (amino sugar, nucleotide sugar metabolism and insulin signaling pathways) and hypoxia response (PI3K-akt, mTOR and MAPK signaling pathways) in the high altitudinal population. The PI3K-akt, mTOR and MAPK pathways modulate the hypoxia-inducible factors, *HIF-1α* and *VEGF* protein expression thus indirectly regulate hypoxia induced angiogenesis, erythropoiesis and vasodilatation. The strategies observed in high altitudinal great tits differ from those described in a closely related species on the Tibetan Plateau, the sedentary ground tit (*Parus humilis*). This species has enhanced selection in lipid-specific metabolic pathways and hypoxia-inducible factor pathway (HIF-1). Comparative population genomics also revealed selection for larger body size in high altitudinal great tits.

Cold temperature and low oxygen level present severe challenges for organisms living at high elevations. The strong selection pressures posed by these harsh conditions have resulted in the evolution of key adaptations for hypoxia resistance, cold tolerance and enhanced metabolic capacity in many mammals and birds^1^,^2^,^3^,^4^,^5^. Despite the fact that the organisms are exposed to similar stresses these high altitude adaptations do not often involve the same genetic mechanisms even in closely related taxa^2^,^3^,^6^,^7^. The differences can possibly be attributed to varying environmental pressures presented in local high altitudinal areas, e.g. in the Andes and the Tibetan Plateau^6^. So far most studies of genomic mechanisms of high altitude adaptations in mammals and birds have focused on sedentary species on the Tibetan Plateau (ca 4,500 m a.s.l.). The results thus describe genetic adaptations to a life under constantly low oxygen and cold conditions. We here investigated if a species that undertakes seasonal altitudinal migrations between environments with variable pressure of oxygen and temperature exhibits genomic adaptations similar to those living under constant conditions.

The great tit (*Parus major*) occupies forest habitats all over the Eurasian continent^8^,^9^. In East Asia, the species occurs mainly in lowland regions yet some populations live at high elevations in eastern Himalayas at the southeastern margin of the Tibetan Plateau. The great tit population in the eastern Himalayas migrates seasonally between their breeding grounds at 4,000 m a.s.l. and their wintering areas at 2,000 m a.s.l.^10^,^11^,^12^. Above 5,000 m, pressure of oxygen (PO2) and temperature are reduced by ~45% and ~33 °C, respectively, compared to that of adjacent sea-level^13^. As oxygen level and temperature vary considerably along the altitudinal gradient^14^, alpine birds face different physiological challenges than if they were living under more constant conditions^15^. It is thus reasonable to predict that the great tits in the eastern Himalayas have developed genetic strategies that differ from if they had lived under constant high altitude conditions. To elucidate the degree and nature of genetic adaptations under varying altitudinal stress we compared the genomics of the great tits in the highland with those living in the lowlands. The genomic mechanisms observed can also be compared with those reported in the closely related ground tit (*Parus humilis*)^5^, the only bird species for which high altitude adaptations have been studied at the genomic level. Unlike the great tit, the ground tit is endemic and sedentary on the Tibetan Plateau at elevations above 4,000 m.

In order to establish the direction of genetic selection, we need to reconstruct the evolutionary origin of the eastern Himalayas great tit population. The great tit is widely distributed in lowland areas^8^,^9^. The observation that the great tits in high altitudinal eastern Himalayas represent an isolated genetic lineage suggests the possibility that population originates from a lowland population in Central Asia^16^. However, this finding is based on a limited amount of mitochondrial DNA and here we used genome-wide data to refine the evolutionary relationships and historical divergence between great tits in the eastern Himalayas and nearby lowlands. We also explored the signature of selection associated with high altitude adaptation.

## Results

### Evolutionary and demographic history

**Figure 1:**
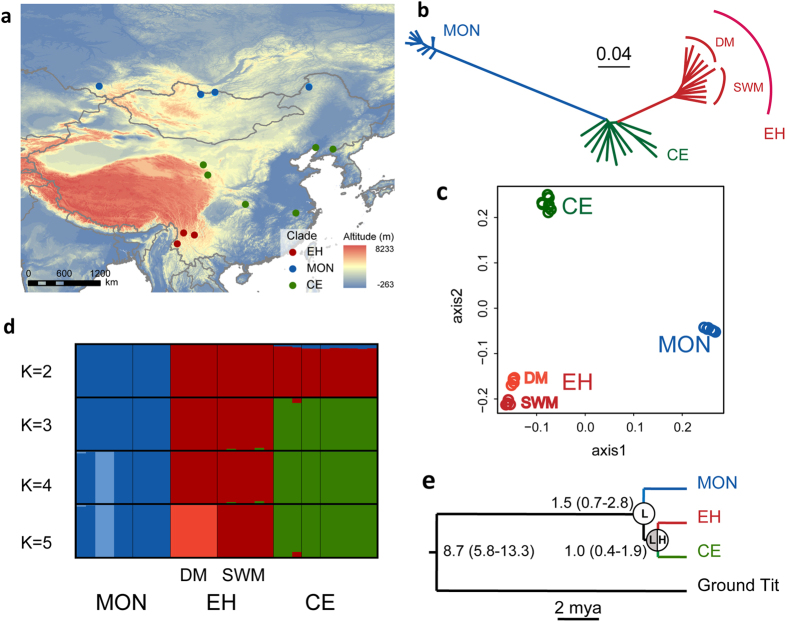
Sampling sites and population genetic structure of the great tit (*Parus major*), red dots indicated individuals belong to the eastern Himalayas group (EH); green dots indicated the Central/East China group (CE); and blue dots indicated the Mongolian group (MON). (**a**) great tit samples from the eastern Himalayas, Central/East China and Mongolia (ArcGIS 9.3, ESRI). (**b**) neighbour-joining tree based on genome-wide SNPs data computed in PHYLIP 3.695. The eastern Himalayas group consisted of individuals from the Diannan Mountains Subregion (DM) and South-West Mountains Subregion (SWM), respectively. (**c**) principal component analysis (PCA) of great tit implemented in GCTA^37^. The plot was based on the first two principal components that explained 43% of the overall variation. **(d**) analysis result of the genetic structure computed in FRAPPE 1.1^38^. The colors in each column represented ancestry proportion over a range of population clusters K2-5. (**e**) phylogenetic tree and divergence times of the three studied groups of great tits based on 179 single-copy orthologous CDs estimated using PAML^39^. Ancestral areas distribution (lowland versus highland) for main internal nodes was estimated by DIVA.

The neighbor-joining tree based on genome-wide data (Supplementary Note 1) recovered the Mongolian population as the most basal lineage and the eastern Himalayas and Central/East China populations as sister lineages (Fig. 1a,b). Within the eastern Himalayas lineage we also found that the great tits in the South-West Mountains Subregion formed a separate group from those in the Diannan Mountains Subregion (Fig. 1b and Supplementary Fig. S1), providing evidence of long-term isolation without gene flow across the eastern Himalayas. The principal component analysis revealed a similar result, with Mongolia, eastern Himalayas and Central/East China lineages as genetically distinct, and a genetic divergence between the South-West Mountains and the Diannan Mountains Subregions in first two PCs, for which PC1 explained 32.36% while PC2 explained 10.66% of the overall variation (Fig. 1c). This population structure was further confirmed using FRAPPE. When K (the number of pre-defined genetic clusters) was set to 2, the Mongolian lineage was clearly separated from the eastern Himalayas and Central/East China lineages, and the latter two lineages were further separated when K was set to 3, suggesting a closer relationship between these than either lineage with the Mongolian lineage (Supplementary Table S1). When K was set to 5, also the substructure within the eastern Himalayas appeared (Fig. 1d). DIVA result confirmed the expectation that the great tits in the eastern Himalayas originated from the nearby lowland (Fig. 1e).

**Figure 2:**
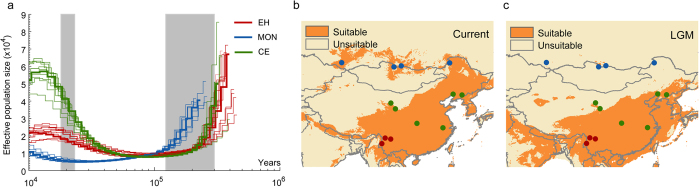
Demographic history of the three groups of the great tit (left) and suitable habitats predicted by ecological niche model during current time and LGM (right). (**a**) Demographic histories of three groups of the great tits inferred using PSMC and further produced in ADOBE ILLUSTRATOR. The periods of the last glacial maximum (LGM; ,0.023–0.018 mya) and the Penultimate glaciation (0.3–0.13 mya) were shaded in grey. **(b**) Palaeodistributions and current distributions for great tit predicted by ecological niche modeling using MAXENT and further produced in ArcGIS. The left panel showed habitats that were predicted to be suitable at the current time and right panel showed the LGM distribution.

The great tits in the eastern Himalayas had a long independent evolutionary history after having been genetically separated from their ancestral populations in the lowland^14^. It was estimated that the Mongolian lineage separated from the others between 0.7 and 2.8 million years ago (mya), and that the eastern Himalayas and Central/East China lineages separated between 0.4 to 1.9 mya (Fig. 1e). Estimates of the population dynamics using a pairwise sequentially Markovian coalescent (PSMC) showed that although all three lineages began to bottleneck around 0.3 to 0.4 mya, the Central/East group started to expand at 0.06 mya, with the effective population size increasing drastically to become six times larger than before. By comparison, the Eastern Himalayas group began to expand at about the same time, 0.06 mya, but has only doubled its effective population size since then. In contrast to these, the Mongolian group experienced a long bottleneck that lasted until 0.02 mya. After this, it slightly increased its effective population size after the last glacial maximum (LGM, 0.023–0.018 mya, Fig. 2a). In agreement with these observations, the suitable habitats of great tit, as predicted by ecological niche modeling, suggested that the local environment in the eastern Himalayas was stable during the Pleistocene glaciations (Fig. 2b).

### Selection associated with high altitude adaptation

**Figure 3:**
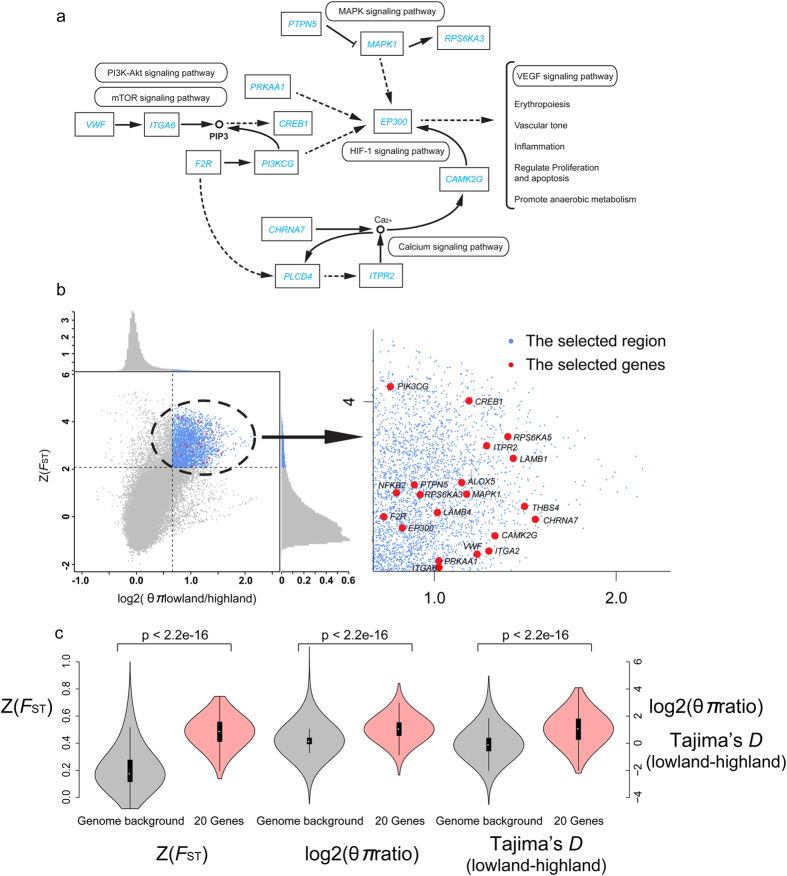
Selection on genes involved in hypoxia response (produced in ADOBE ILLUSTRATOR). (**a**) these genes involved in MAPK signaling pathway (map 04010), PI3K-akt signaling pathway (map 04151), mTOR signaling pathway (map 04150) and calcium signaling pathway (map 04020). Abbreviations, annotations and connections were presented in accordance with KEGG standards, where solid lines indicated direct relationships between genes and dashed lines indicated that more than one step were involved in the process. Not all genes under selection were shown in here, see Table 1 for more details. (**b**) Distribution of θπ ratio (θπ, lowland/θπ, highland) and *F*_ST_ values, which were calculated in 100-kb windows sliding in 10-kb steps (produced in PLOT in R package). Data points in blue (corresponding to the 5% θπ ratio distribution, where θπ was 1.58, and the 5% *F*_ST_ distribution, where *F*_ST_ was 0.425) were regions under selection in highland great tits. The genomic regions contained 20 hypoxia genes in above pathways were marked in red. (**c**) The 20 hypoxia genes exhibited a higher *F*_ST_, θπ ratio and Tajima’s *D* lowland—Tajima’s *D* highland compared with the genome background by Student’s t-tests (T.TEST in R package).

We measured genome-wide variation between the eastern Himalayas group and the lowland group (Mongolian and Central/East China groups) to detect signatures of selection associated with high altitude. In the eastern Himalayas group the screen for selective sweep detected 183 genes (Supplementary Table 2) under selection, which were primarily involved in metabolic processes and hypoxia response (Supplementary Table S3). The enriched metabolism pathways included amino sugar and nucleotide sugar metabolism (5 genes, P < 0.05) and insulin signaling pathway (11 genes, P < 0.023). Hypoxia response genes were enriched in the MAPK signaling pathway, the PI3K-akt signaling pathway, the mTOR signaling pathway and the calcium signaling pathway (Fig. 3a and Table 1). After we carried out individual analysis for each candidate hypoxic gene, twenty genes were identified showing evidence of divergent selection specific to the eastern Himalayas group with a marked divergence from the Mongolian and Central/East groups (Fig. 3b and Supplementary Fig. S2). These genes exhibited higher *F*_ST_, higher θπ ratios, and higher (Tajima’s *D* for lowland great tits—Tajima’s *D* for high altitude great tits) values compared to those of the genomic background (Fig. 3c and Supplementary Table S4). A detailed description of these genes and their relevance to hypoxic adaptation was presented in the Supplementary Note 2. We further selected four candidate genes with amid acid substitutions (*LAMB4*, *THBS4*, *ITGA2* and *F2R*) to validate SNPs associations with altitude distribution. Between 16 and 32 additional great tit individuals were Sanger sequenced and it was confirmed that the observed amid acid substitutions were nearly fixed in the eastern Himalayas great tits and strongly correlated with their high altitude distribution (Supplementary Fig. S3).

We also identified signals of selection in developmental genes related to morphogenesis (regulation of actin cytoskeleton, KEGG map 04810; adherens junction, KEGG map 04520) among great tits in the eastern Himalayas, Central/East and Mongolia. Congruent with this result, we observed that the three groups of great tits differed in their average size: the Mongolian great tits had the largest body size, tail, tarsus and bill lengths, the eastern Himalayas great tits were of intermediate sizes and the Central/East great tits were the smallest (see Supplementary Fig. S4 and Table S5).

## Discussion

### Evolutionary and demographic history

The ecological stratification and environmental heterogeneity of many mountain regions have greatly promoted speciation and diversification in birds and other organisms^17^. In the case of the great tit the population in the eastern Himalayas has diverged genetically from those in the lowland. Comparative population genomics also detects an even younger differentiation within the eastern Himalayas with individuals in the South-West Mountains Subregion differing genetically from those in the Diannan Mountains Subregion. This was not observed in a previous study of the population structure in the great tit^16^ (using mitochondrial DNA data), but could be detected through the increased genomic resolution provided by next generation sequencing data^18^. Similar patterns have been observed in four other bird species inhabiting the eastern Himalayas, green-backed tit (*Parus monticolus*), black-throated bushtit (*Aegithalos concinnus*), grey-cheeked fulvetta (*Alcippe morrisonia*) and red-headed tree babbler (*Stachyridopsis ruficeps*)^19^. In all of these species the different ecological subregions of the eastern Himalayas act as geographical barriers preventing gene flow between populations within and outside of the mountainous areas, leading to long-term isolation and *in situ* diversification^19^. During the Pleistocene, while other regions were heavily influenced by the glacial cycles (as suggested by the drastically fluctuating population size in Central/China and the decreasing habitats in Mongolia), the eastern Himalayas was climatically stable^20^,^21^,^22^. This allows the great tit population to persist in this montane environment for a long time and to evolve high altitude adaptations.

### High altitude adaptation in energy metabolism

The great tits in the eastern Himalayas have a long independent evolutionary history after they diverges from the lowland populations. Since their isolation, they have been exposed to different forces of selection in their local montane environment compared to their lowland ancestors. Our results show that the great tits in the eastern Himalayas cope with their high altitude living condition by evolving adaptations to increase energy metabolism and hypoxia response. Although these adaptations are similar to those reported in the closely related ground tit^5^, they do differ in important ways. Both species have increased their energy metabolism but they utilize different optimal-fuel strategies. The positively selected genes in the ground tit are mostly related to fatty-acid metabolic pathways (Supplementary Table S6), whereas those in the great tit are related to the carbohydrate metabolic pathways, such as amino sugar, nucleotide sugar and insulin metabolism. The ability to effectively allocate fuel substrates for oxidative metabolism is critical at high elevation. Compared to the oxidation of carbohydrates, the oxidation of lipids has a higher energy density and can elevate the thermogenic capacity for a prolonged period of cold exposure^15^. Lipids make up more than 80% of the total energy reserves of animals^15^. The ground tit is sedentary on the Tibetan Plateau and thus forced to cope with the cold and hypoxic environment year-round making high energy lipids the preferred source for fuel. Carbohydrates may be a better choice for great tits in the eastern Himalayas that seasonally migrate between 2,000 and 4,000 m a.s.l. Carbohydrates can be rapidly depleted under prolonged cold conditions, but their oxidation yields about 15% more ATPs per mole of oxygen than for lipids^23^. This stoichiometric advantage can provide a better strategy when living at medium elevations^15^. Also the seasonal altitudinal migration of the eastern Himalayas’ great tits makes it better to rely on energy-effective carbohydrates as the optimal energy expenditure instead of lipids. Despite being rather closely related phylogenetically, the great tit and ground tit have evolved different genetic mechanisms in coping with their high altitude life.

### High altitude adaptation in hypoxia response

The great tits in the eastern Himalayas show strong positive selection in genes related to hypoxia response. While most genomic studies on sedentary species (also ground tit) on the Tibetan Plateau have showed evidence of positive selection on genes involved in the HIF-1 and VEGF pathways^1^,^2^,^3^,^4^,^5^, a majority of the genes (19 out of 20 genes) showing selection for hypoxia response in great tits are involved in the PI3K-akt, mTOR and MAPK signaling pathways (Table 1). Although the HIF-1 and VEGF signaling pathways are directly involved in angiogenesis, erythropoiesis, vasodilatation and glucose metabolism in response to hypoxia^24^,^25^, the HIF-1α and VEGF protein expression are modulated by the PI3K-akt, mTOR and MAPK signaling pathways^25^,^26^,^27^. Under hypoxic conditions, cells increase VEGF expression through the PI3K-dependent pathways (especially PI3K-akt, MAPK, and mTOR). These pathways also regulate HIF-1α protein expression. Specifically, the glycation end product induced HIF-1 activation is dependent on MAPK, whereas insulin-induced HIF-1 activation is dependent on PI3K-akt^25^,^27^. Additionally, genes in the calcium signaling pathway are also detected to be under selection in the high altitude great tits. Calcium transporters are very sensitive to oxygen deficiency^28^. One of the constant early responses to hypoxia in almost all cell types is an increase in intracellular Ca2 + . Therefore, MAPK, PI3K-akt, mTOR and calcium signaling pathways may constitute a network that regulates gene expression in the high altitudinal great tits.

### High altitude adaptation in morphology

Selection in genes involved in the development is also found to be strong in the great tits in the eastern Himalayas compared to those in Central/East China. This may be explained by, on average, the larger body size, longer wing and tarsus lengths in great tits living at high altitude. The larger size and heavier body mass are probably advantageous in heat retention. Furthermore, body size is often correlated with hemoglobin O2 affinity, as larger animals tend to have higher O2 affinity^29^, which is likely a thermoregulatory adaptation to the colder climate at high elevations. This observation is in analogy with the fact that the high altitude ground tit is larger than any other species of tits^5^. However, we also observed an accelerated rate of developmental genes in the Mongolian great tits. This population also has a larger average body size, but in this case the large size has evolved in response to another selection pressure. The Mongolian great tits predominantly live in deciduous forests, in contrast to the populations in the eastern Himalayas and Central/East China that inhabit in coniferous forest habitats^8^. It has been shown that great tits living in deciduous forests are larger on average than those living in coniferous forests because the former habitats provide a larger supply of food resources^30^,^31^.

## Materials and Methods

### Study area and sampling

Tissue samples were obtained from 32 great tits from 13 localities, including 11 individuals from the eastern Himalayas, 11 from Central/East China and 10 from Inner Mongolia and Mongolia (Fig. 1a and Supplementary Table S7). The eastern Himalayas region consists of several zoogeographic subregions^11^,^32^ of which our sampling covered two, the South-West Mountain and the Diannan Mountain Subregions (Supplementary Fig. S1). In contrast to the alpine landscape of the eastern Himalayas, the Central/East China was generally lowland^33^. The samples were preserved in 100% ethanol and kept in the collection of the National Zoological Museum, Institute of Zoology, Chinese Academy of Sciences. All used samples were unprotected bird specimens from the specimen collection of the National Zoological Museum, Institute of Zoology, Chinese Academy of Sciences (address: No1 Beichen West Road, Chaoyang District, Beijing, China). The collection was under the permit from Forestry Department and conformed to the National Wildlife Conservation Law in China. No living animal experiments were conducted in the current research.

### Sequencing and data production

#### Sequencing strategy

Genomic DNA was extracted from muscle samples. All samples were sequenced on the Illumina sequencing platform (HiSeq 2000) in *Novogene Bioinformatics Institute* (Beijing, China). DNA libraries (500 bp) were constructed according to the manufacturer’s introductions (Illumina). Using a whole genome shotgun strategy we generated a total of 197.84 Gb of paired-end reads of 100 bp length.

#### Sequence quality checking and filtering

To avoid reads with artificial bias in the process of library construction and sequencing (i.e. low quality reads, which mainly resulted from base-calling duplicates and adapter contamination), we carried out quality control and filtered out sequences according to the following criteria:

(a) any reads with ≥10% unidentified nucleotides (N);

(b) any reads with >10 nt aligned to the adapter sequence, allowing ≤10% mismatches;

(c) any reads with >50% bases having phred quality <5;

(d) putative PCR duplicates generated by PCR amplification in the library construction process (i.e. two paired-end reads were the same).

A total of 186.32 Gb (94.18%, out of 197.84 Gb) high quality paired-end reads was retained for the further analyses (Supplementary Table S8).

### Read mapping and SNPs calling

After quality controls the reads were mapped to the ground tit genome using BWA^34^, with parameters: aln −o 1 −e 10 −t 4 −l 32 −i 15 −q 10, and reads having a mean of approximately 5x depth for each individual and >90% coverage of the ground tit genome were retained for SNP calling. We applied a Bayesian algorithm based SAMtools program to call SNPs using command ‘mpileup’ with parameters as ‘−q 1 −C 50 −S −D −m 2 −F 0.002 −u’. Meanwhile, we calculated the genotype likelihoods from reads for each individual at every genomic location and estimated the allele frequencies. We filtered SNPs using VCFtools^35^ and GATK^36^ by following criteria: a) coverage depth > = 4 and < = 1000; b) root mean square (RMS) mapping quality > = 20; c) the distance of adjacent SNPs > = 5 bp; d) the distance to a gap > = 5 bp; e) reads quality value > = 30. We further filtered all homogeneous SNPs of the great tits to remove the SNPs between great tit and ground tit, an unavoidable result due to the fact that the SNPs call was based on the ground tit genome. After filtering a total of 3,613,365 high quality SNPs was retained.

### Population structure and evolutionary history

To carry out population structure and selection analyses, we removed all SNPs with a minor frequency (MAF) < = 0.1 and kept only SNPs that occurred in more than 90% of the individuals. We used all high quality SNPs to infer population structure. To estimate phylogenetic relationships the pairwise genetic distances were calculated among all samples to generate a neighbor-joining (NJ) tree using PHYLIP 3.695 (http://evolution.Genetics.Washington.edu/phylip.html). We performed a principal component analysis (PCA) using the package GCTA^37^. A Tracy–Widom test was used to determine the significance level of the eigenvectors. Population genetic structure was further inferred using FRAPPE 1.1^38^. To explore the divergence of great tit individuals, we set the pre-defined genetic clusters (K) from 2 to 5 to cover the maximum numbers of lineages that could be identified in the mtDNA trees^16^. We run the analysis with 10,000 maximum iterations.

We also inferred a group-level phylogeny using the MCMC tree program implemented in PAML^39^ based on 179 single-copy coding DNA sequences (CDs). The ground tit genome was used for homology search these CDs by TBLASTN. Only the CDs with >4x depth were identified for each great tit individual and these CDs were subsequently aligned to group-level consensus sequences. We estimated the divergence time using a timeframe of 9.0 (5.9 to 13.5) mya between the ground tit and great tit^5^. To further infer the evolutionary origin of the eastern Himalayas great tits, we reconstructed ancestral distributions of the group-level phylogeny using dispersal-vicariance analysis in DIVA 1.1^40^.

### Pleistocene demographic history and stable refugia

The demographic history of the three great tit groups was inferred by a pairwise sequentially Markovian coalescence (PSMC) model. As all samples were sequenced at 5x depth, we used all individuals in each group to infer population demographic history to get a consistent result. Parameters were set as ‘−N30 −t15 −r5 −p 45*2’. The mutation rate was set to be 2.78 × 10^−9^ per generation, which was estimated by dividing the sequence divergence between the ground tit and great tit by the estimated time for their split (9.0 mya). The generation time for the great tit was set at 1.5 years^10^.

We reconstructed the palaeodistributions and current distributions for great tits in the eastern Himalayas, Central/East China and Mongolia. Ecological niche models predicted the current distribution in MAXENT^41^ using 19 climatic variables at 2.5-min resolution (WorldClim data set)^42^. The models were subsequently applied to climatic variables generated from Community Climate System Model (CCSM) to estimate suitable habitats during the LGM (0.018–0.023 mya)^43^,^44^.

### Identification of selected regions

To detect signatures of selection associated with high altitudes, we measured genome-wide variation between the highland (eastern Himalayas) and lowland groups (Mongolia and Central/East China). We applied a sliding window approach (100-kb window sliding with a step size of 10-kb) to identify selective regions associated with high altitude. Genomic regions with significant high *F*_ST_ statistic (corresponding to a top 5% level, where *F*_ST_ was 0.425) and θπ ratio (θπ, lowland/θπ, high altitude, a top 5% level where θπ ratio was 1.58) values were identified as highly divergent.

### Annotation analysis of selection regions

We annotated genes in selected genomic regions using the ground tit genome and a total of 183 genes were identified to be under positive selection in eastern Himalayas’ great tits. These genes were submitted to Gene Ontology and KEGG databases for enrichment analyses. A false discovery rate (FDR) corrected binomial distribution probability approach was used to test significant enriched gene function at a level of *P* < 0.05^45^.

### Targeted gene analysis

For target gene analyses, we used a higher resolution sliding window analysis in 10 kb window (compared 100 kb window in Selection analysis) to calculate *F*_ST_, θπ and Tajima’s *D* between high altitude and lowland great tits (Supplementary Fig. S2). We compared *F*_ST_, θπ and Tajima’s *D* values of these genes with those of whole genome level by t-tests (Table S3). For each gene, SNPs from CDs of these genes were extracted to infer neighbor-joining trees using TreeBeST^46^.

### Biometrical measurement of three groups of the great tit

We compared biometrical measurements made on fifty study skins in the National Zoological Museum, Institute of Zoology, Chinese Academy of Sciences, including 13 specimens from Mongolia and Inner Mongolia, 18 from the eastern Himalayas and 19 from Central/East China. The lengths of the wing, tail, tarsus and bill were measured using a digital caliper. Body masses and body lengths were taken from the field collection records. Differences in the measurements between the three groups of the great tits were analyzed using a one-way analysis of variance (ANOVA, SPSS 20).

## Additional Information

**Accession codes**: Sequencing data for the great tit have been deposited in Short Read Archive under project number PRJNA SRP274877.

**How to cite this article**: Qu, Y. *et al.* Genetic responses to seasonal variation in altitudinal stress: whole-genome resequencing of great tit in eastern Himalayas. *Sci. Rep.* **5**, 14256; doi: 10.1038/srep14256 (2015).

## Supplementary Material

Supplementary Information

## Figures and Tables

**Table 1:** Candidate genes involved in hypoxia response showing selection in the eastern Himalayas great tits.

**Pathways**	**Kegg map**	**Gene**	**Hypoxia response in other organisms**	**Reference**
MARK signaling pathway	Map 04010	*NFKB2*	Hypoxia-induced cell	25,47,48,49
		***MARK1***	Hypoxia-induced cell	25
		***RPS6KA3***		
		*PTPN5*		
		*RPS6KA5*		
PI3K-akt signaling pathway	Map 04151	*CREB1*	Hypoxia-induced cell	49
		***F2R***		
		*LAMB4*		
		*LAMB1*		
		*ITGA6*	Hypoxia-induced cell	50
		*ITGA2*	Tibetan pig	51
		***PI3KCG***	Tibetan	52
		***PRKAA1***	Andeans	53
		*THBS4*	Hypoxia-induced mouse	54
		*VWF*	Hypoxia-induced cell	55
mTOR signaling pathway	Map 04150	***PI3KCG***	Tibetan	52
		***PRKAA1***	Andeans	1
		***RPS6KA3***		
Calcium signaling pathway	Map 04020	*CHRNA7*		
		***F2R***		
		*ITPR2*	Hypoxia-induced cell	56,57
		***CAMK2G***		
HIF-1 signaling pathway	Map 04066	*EP300*	Hypoxia-induced cell	58
		***PI3KCG***	Tibetan	52
		***CAMK2G***		
		***MARK1***	Hypoxia-induced cell	25
VEGF signaling pathway	Map 04370	***PI3KCG***	Tibetan)	52
Other genes		*ALOX5*	Human	59

Bold showed genes sharing between different pathways.
