# Supplementary material for: Genetic responses to seasonal variation in altitudinal stress: whole-genome resequencing of great tit in eastern Himalayas
Source: Sci Rep. 2015 Sep 25;5:14256. doi: 10.1038/srep14256 (PMC4585896; doi:10.1038/srep14256)
Supplement: Supplementary Information [file srep14256-s1.pdf]

Supplementary information

Genetic responses to seasonal variation in altitudinal stress: whole-genome resequencing of great tit in eastern Himalayas

Yanhua Qu[1,4], Shilin Tian[2,4], Naijian Han[1], Hongwei Zhao[2], Bin Gao[1], Jun Fu[2], Yalin Cheng[1], Gang Song[1], Per G. P. Ericson[3], Yong E. Zhang[1], Dawei Wang[2], Qing Quan[1], Zhi Jiang[2], Ruiqiang Li[2], Fumin Lei[1*]

1. Key Laboratory of Zoological Systematics and Evolution, Institute of Zoology, Chinese Academy of Sciences, Beijing 100101, China.
2. Novogene Bioinformatics Institute, Beijing 100083, China
3. Department of Vertebrate Zoology, Swedish Museum of Natural History, PO Box 50007, SE-10405 Stockholm, Sweden.
4. These authors contributed equally to this work.

* Corresponding author

Email: leifm@ioz.ac.cn (F.L.)

Supplementary Notes

Supplementary Note 1. SNPs genetic diversity.
Supplementary Note 2. Description of genes relevance to hypoxia adaptation.

Supplementary Tables

Supplementary Table S1. Pairwise F_{ST} between the three great tit groups.
Supplementary Table S2. 183 genes showed signal of positive selection in the eastern Himalayas great tits.
Supplementary Table S3. Functional gene categories enriched for the high altitude great tits (the eastern Himalayas) compared to lowland great tits (Central/East China and Mongolia).
Supplementary Table S4. 20 hypoxia genes showed signatures of selection in high altitude great tits.
Supplementary Table S5. Biometrical measurement of three great tit groups.
Supplementary Table S6. Positive selection in the fatty-acid metabolism pathways of ground tit (*Parus humilis*).
Supplementary Table S7. Sampling information of the great tits used in this study.
Supplementary Table S8. Statistics of reads mapping and coverage.
Supplementary Table S9. Statistics of SNPs in whole genome of great tit.

Supplementary Figures

Supplementary Figure S1. Sampling sites of the great tit and ecological subregions within the eastern Himalayas (ArcGIS 9.0, ESRI). Our sampling in the eastern Himalayas covered two zoogeographic subregions, the South-West Mountain Subregion and the Diannan Mountain Subregion.

Supplementary Figure S2. Divergent selection on the 20 hypoxia response genes. (a) gene trees of *ALOX5*, *CAMK2G*, *CHRNA7*, *CREB1*, *EP300*, *F2R*, *ITGA2*, *ITGA6*, *ITPR2*, *LAMB1*, *LAMB4*, *MAPK1*, *NFKB2*, *PIK3CG*, *PRKAA1*, *PTPN5*, *RPS6KA3*, *RPS6KA5*, *THBS4* and *VWF* estimated using TreeBeST. Red, the eastern Himalayas great tits; green, the Central/East China great tits; blue, the Mongolian great tits. (b) genes showing strong selective signals in the eastern Himalayas great tits. F_{ST} values, $\theta\pi$ ratios and Tajima's D values were calculated using a 10-kb sliding window. Genomic regions located above the upper horizontal dashed line in F_{ST} plot (corresponding to a 5% significance level of values, where F_{ST} was 0.425) and $\theta\pi$ ratio plot (a 5% significance level of the $\theta\pi$ ratio, where the $\theta\pi$ ratio is 1.58) were termed as regions with signals for strong selective sweep in high altitude great tits (gray regions). The green lines in the Tajima's D plots represent great tits in the eastern Himalayas and blue lines great tits in Mongolia and Central/East China. Genome annotations were shown at the bottom (black bar, Coding DNA sequences (CDs); red bar, genes). The boundaries of these genes were marked in blue.

Supplementary Figure S3. Sequence assemblies of four genes (*LAMB4*, *THBS4*, *ITGA2* and *F2R*) for which between 16 to 32 individuals of great tits have been Sanger sequenced. The selection analysis confirmed that observed amid acid substitutions were nearly fixed in the eastern Himalayas population.

Supplementary Figure S4. Box plots of the biometrical measurement of three groups of the great tits plotted using GGPLOT 2 [6] in R 3.1.1 [7].

Supplementary Note 1. SNPs genetic diversity

Whole genome resequencing of 32 great tits (Fig. 1a and Supplementary Table S7) generated a total of 197.84 Gb data (100 bp paired-end reads). The raw reads were processed by Illumina HiSeq control software and resulted in 186.32 Gb of clean data, mostly to an approximately 5x depth and >90% coverage (Supplementary Table S8). We identified a total of 3,613,365 SNPs for subsequently analysis. The numbers of SNPs in the great tits in the eastern Himalayas, Central/East China and Mongolia were 2,999,524, 3,093,290 and 2,237,921, respectively (Supplementary Table S9). The levels of genome-wide genetic diversity were almost twice as high in the eastern Himalayas (5.19×10^{-3}) and Central/East China groups (5.30×10^{-3}) as in the Mongolian group (3.15×10^{-3}).

Supplementary Note 2. Description of genes relevance to hypoxia adaptation

Previous studies of genome scans in organisms on the Tibetan plateau and other high altitudinal environments have independently identified genes that participate in these pathways, e.g., *PI3KCG* and *PRAAA1* in Tibetans and Andeans [1-2] and *ITGA2* in Tibetan pig [3]. Furthermore, hypoxia-induced experiments in laboratory cells and mice also provided evidence for hypoxia response of other genes (Table 1). *EP300* is critical co-activator of the HIF-1 pathway [4-7]. A blockage of the interaction of *EP300* with HIF-1α attenuates hypoxia-inducible transcription in cell culture [7]. *ITGA6* activates HIF-1α under the hypoxia condition and regulates VEGF transcription [8]. *PI3KCG* and *CREB1* in PI3K-akt pathway are important regulators of HIF-1α expression, HIF-1 activation and VEGF expression during prolonged hypoxia [9-10]. *MAPK1* activates HIF-1 by promoting the phosphorylation of *HIF-1α* and its accumulation in response to advanced glycation end products or mersalyl [11-12]. *VWF* is a glycosylated protein expressed exclusively in endothelial cells and hypoxia significantly increases *VWF* expression in heart, lung and liver [13].

Positively selected genes in the calcium signaling pathway were also detected in high altitudinal great tits. Calcium transporters are very sensitively to oxygen deficiency. One of the constant early responses to hypoxia in almost all cell types is to increase intracellular Ca2+ ([Ca2+]i). *ITPR2* and *PLCD4* in calcium pathway provide a link to the hypoxia-activated mechanism of ([Ca2+]i) increase and releases of Ca2+ from intracellular Ca2+ stores [14]. These changes are mediated with increased activity of Ca2+ ATPase and increased number of IP3 receptors (*IP3R*) on nuclear envelope and membranes of endoplasmic reticulum [15].

Additionally, we also detected other genes related to hypoxia response but not involved directly in the pathways mentioned above, for example *ALOX5*, which catalyzes the first step in leukotriene biosynthesis and plays a role in inflammatory processes. A suppression of *ALOX5* activity reduces the cell proliferation and induces intrinsic mitochondrial apoptotic pathway [16].

References:

1. Bigham, A. *et al.* Identifying signatures of natural selection in Tibetan and Andean populations using dense genome scan data. *PloS Genetics* 6, e1001116 (2010).
2. Zhao, R., Feng, J. & He, G. MiR-613 regulates cholesterol efflux by targeting LXR alpha and ABCA1 in PPAR gamma activated THP-1 macrophages. *Biochemical and biophysical research communications* 448, 329–334 (2014).
3. Ai, H., Yang, B., Li, J., Xie, X., Chen, H. & Ren, J. Population history and genomic signatures for high-altitude adaptation in Tibetan pigs. *BMC genomics* 15, 834 (2014).
4. Arany, Z. *et al.* An essential role for p300/CBP in the cellular response to hypoxia. *Proc. Natl. Acad. Sci. USA* 93, 12969–12973 (1996).
5. Carrero, P., Okamoto, K., Coumailleau, P., O'Brien, S., Tanaka, H. & Poellinger, L. Redox-regulated recruitment of the transcriptional co-activators CREB-binding protein and SRC-1 to hypoxia-inducible factor 1α. *Mol. Cell Biol.* 20, 402–415 (2000).

6. Ema, M. *et al.* Molecular mechanisms of transcription activation by HIF and HIF1α in response to hypoxia: their stabilization and redox signal-induced interaction with CBP/p300. *EMBO. J.* 18, 1905–1914 (1999).
7. Kung, A.L. *et al.* Small molecule blockade of transcriptional coactivation of the hypoxia-inducible factor pathway. *Cancer Cell* 6, 33–43 (2004).
8. Chuang, J., Yoon, S., Datta, K., Bachelder, R.E. & Mercurio, A.M. Hypoxia-induced vascular endothelial growth factor transcription and protection from apoptosis are dependent on alpha-6-beta-1 integrin in breast carcinoma cells. *Cancer Res.* 64, 4711–4716 (2004).
9. Nakayama, K. cAMp-response element-binding protein (CREB) and NR-KB transcription factors are activated during prolonged hypoxia and cooperatively regulate the induction of matrix metalloproteinase MMP1. *J. Bio. Chem.* 288, 22584–22595 (2013).
10. Stiehl, D.P., Wirthner, R., Köditz, J., Spielmann, P., Camenisch, G. & Wenger, R.H. Increased prolyl 4-hydroxylase domain proteins compensate for decreased oxygen levels. Evidence for an autoregulatory oxygen-sensing system. *J. Biol. Chem.* 281, 23482–23491 (2006).
11. Minet, E. *et al.* ERK activation upon hypoxia:involvement in HIF-1 activation. *FEBS Letter* 468, 53–58 (2000).
12. Treins, C., Giorgetti-Peraldi, S., Murdaca, J., Semenza, G.L. & Obberghen, E.V. Insulin stimulates hypoxia-inducible factor 1 through a phosphatidylinositol 3-Kinease/Target of Rapamycin-dependent signaling pathway. *Journal of Biological Chemistry* 277, 27975–27981 (2002).
13. Mojiri, A. *et al.* Hypoxia results in upregulation and De novo activation of von willebrand factor expression in lung endothelial cells. *Vascular Biology* 33, 1329–1338 (2013).
14. Toescu, E.C., Verkhratsky, A. & Landfield, P.W. Ca2+ regulation and gene expression in normal brain aging. *Trends Neurosci.* 27, 614–620 (2004).
15. Jurkovicova, D. *et al.* Hypoxia differently modulates gene expression of Inositol 1, 4, 5-Trisphosphate receptors in Mouse kidney and HEK 293 cell line. *Annals of the New York Academy of Sciences* 1148, 421–427 (2008).
16. Bishayee, K. & Khuda-Bukhsh, A.R. 5-lipoxygenase antagonist therapy: a new approach towards targeted cancer chemotherapy. *Acta. Biochim. Biophys. Sin.* 45, 709–719 (2013).

Supplementary Table S1. Pairwise F_{ST} between the three great tit groups.

	Mongolia	Eastern Himalayas	Central/East China
Mongolia			
Eastern Himalayas	0.5028		
Central/East China	0.4357	0.2335	

Supplementary Table S2. 183 genes showed signal of positive selection in the eastern Himalayas great tits.

Genes	SWISS
Evm.model.scaffold15230.48	WNT8B
Evm.model.scaffold16291.37	WNT6
Evm.model.scaffold16291.38	WNT10A
Evm.model.scaffold21931.16	WIF1
Evm.model.scaffold22289.5	VWF
Evm.model.scaffold15230.11	VCL
Evm.model.scaffold23087.6	UTP15
Evm.model.scaffold17397.19	UGDH
Evm.model.scaffold23041.2	UGCG
Evm.model.scaffold20308.11	UBE2N
Evm.model.scaffold22308.1	TYRP1
Evm.model.scaffold1821.9	TUBA4A
Evm.model.scaffold23068.33	TOP3B
Evm.model.scaffold21641.14	THBS4
Evm.model.scaffold23044.18	TH
Evm.model.scaffold19776.37	TGFB3
Evm.model.scaffold1821.8	TBA5
Evm.model.scaffold22671.18	SUV420H1
Evm.model.scaffold17474.9	SRP54
Evm.model.scaffold22997.4	SPTLC1
Evm.model.scaffold13364.18	SOCS1
Evm.model.scaffold12377.16	SNX29
Evm.model.scaffold10071.19	SNW1
Evm.model.scaffold12228.27	SMS
Evm.model.scaffold16291.25	SLC11A1
Evm.model.scaffold21181.7	SHC3
Evm.model.scaffold22820.2	SH3GL2
Evm.model.scaffold15230.47	SEC31B
Evm.model.scaffold21181.8	S1PR3
Evm.model.scaffold16291.29	RQCD1
Evm.model.scaffold3693.14	RPS6KA5
Evm.model.scaffold12228.21	RPS6KA3
Evm.model.scaffold19805.5	RPS6
Evm.model.scaffold21284.27	RPL5
Evm.model.scaffold17475.4	RPL37
Evm.model.scaffold13364.19	RMI2
Evm.model.scaffold23040.6	RHOA
Evm.model.scaffold23034.11	RFK
Evm.model.scaffold2701.61	RBX1
Evm.model.scaffold21898.7	RBPJ
Evm.model.scaffold1954.1	RAD51B
Evm.model.scaffold16315.4	PTPRB
Evm.model.scaffold21896.26	PTPN5
Evm.model.scaffold1821.28	PSMD14
Evm.model.scaffold12566.49	PSMC3
Evm.model.scaffold22850.2	PSD3
Evm.model.scaffold15230.40	PSD
Evm.model.scaffold8566.11	PRKCE
Evm.model.scaffold8566.12	UNKNOWN
Evm.model.scaffold22742.12	PRKAR2A
Evm.model.scaffold16291.35	PRKAG3
Evm.model.scaffold17475.5	PRKAA1
Evm.model.scaffold15230.22	PPP3CB

Evm.model.scaffold21898.17	PPARGC1A
Evm.model.scaffold22269.35	POLR2F
Evm.model.scaffold22637.2	PLK2
Evm.model.scaffold16291.30	PLCD4
Evm.model.scaffold22269.30	PLA2G6
Evm.model.scaffold23034.9	PIP5K1B
Evm.model.scaffold14622.13	PIP4K2A
Evm.model.scaffold4277.3	PIK3CG
Evm.model.scaffold1954.4	PIGH
Evm.model.scaffold23000.1	PIGG
Evm.model.scaffold23063.7	PELO
Evm.model.scaffold23000.4	PDE6B
Evm.model.scaffold12020.3	PAIP1
Evm.model.scaffold22026.2	OXCT1
Evm.model.scaffold22087.33	OGG1
Evm.model.scaffold22930.1	NTRK2
Evm.model.scaffold22936.2	UNKNOWN
Evm.model.scaffold22289.7	NTF3
Evm.model.scaffold10071.16	NRX3A
Evm.model.scaffold3737.6	NRG1
Evm.model.scaffold12020.2	NNT
Evm.model.scaffold23067.4	NLN
Evm.model.scaffold15230.39	NFKB2
Evm.model.scaffold15230.46	NDUFB8
Evm.model.scaffold19995.5	NCBP1
Evm.model.scaffold17157.5	NANS
Evm.model.scaffold15335.13	NAMPT
Evm.model.scaffold22700.4	NAA38
Evm.model.scaffold22654.35	MUTYH
Evm.model.scaffold23044.15	MRPL23
Evm.model.scaffold23063.4	MOCS2
Evm.model.scaffold23016.53	MLTK
Evm.model.scaffold19776.26	MLH3
Evm.model.scaffold23068.31	MAPK1
Evm.model.scaffold23016.26	LRP2
Evm.model.scaffold22850.1	LPL
Evm.model.scaffold23089.1	LPAR1-A
Evm.model.scaffold17157.1	LNPEP
Evm.model.scaffold17397.18	LIAS
Evm.model.scaffold17991.20	LGMN
Evm.model.scaffold4763.20	LAMB4
Evm.model.scaffold4763.21	LAMB1
Evm.model.scaffold21842.6	KCNMA1
Evm.model.scaffold21953.12	ITPR2
Evm.model.scaffold23016.48	ITGA6
Evm.model.scaffold23063.5	ITGA2
Evm.model.scaffold23063.6	ITGA1
Evm.model.scaffold20994.8	IQGAP1
Evm.model.scaffold23044.17	INS
Evm.model.scaffold22064.11	INHBB
Evm.model.scaffold21152.1	IL6ST
Evm.model.scaffold21641.10	HOMER1
Evm.model.scaffold23087.10	HEXB
Evm.model.scaffold22010.3	GZMA
Evm.model.scaffold12377.15	GSPT2
Evm.model.scaffold23040.8	GRM2
Evm.model.scaffold17157.7	GNE

Evm.model.scaffold23034.16	GNAQ
Evm.model.scaffold23087.14	GCNT4
Evm.model.scaffold21079.19	GCLC
Evm.model.scaffold15230.37	GBF1
Evm.model.scaffold21243.18	GAS1
Evm.model.scaffold21181.1	GADD45G
Evm.model.scaffold2983.2	GAB1
Evm.model.scaffold23042.19	FZD7
Evm.model.scaffold23042.28	FZD5
Evm.model.scaffold23063.1	FST
Evm.model.scaffold19000.1	FREM1
Evm.model.scaffold3737.3	FNTA
Evm.model.scaffold20849.1	FGF10
Evm.model.scaffold19776.22	FCF1
Evm.model.scaffold23040.3	FANCD2
Evm.model.scaffold20994.6	F2RL1
Evm.model.scaffold20994.7	F2R
Evm.model.scaffold2701.60	EP300
Evm.model.scaffold21079.20	ELOVL5
Evm.model.scaffold19776.25	EIF2B2
Evm.model.scaffold14622.9	DNAJC1
Evm.model.scaffold1821.10	DNAJB2
Evm.model.scaffold19995.10	DNAJA1
Evm.model.scaffold23090.1	DNAI1
Evm.model.scaffold21641.7	DMGDH
Evm.model.scaffold12566.14	DLL4
Evm.model.scaffold22860.2	DDX58
Evm.model.scaffold17475.7	DAB2
Evm.model.scaffold16291.36	CYP27A1
Evm.model.scaffold298.3	CYB5R3
Evm.model.scaffold21243.16	CTSL1
Evm.model.scaffold12566.28	CREB3L1
Evm.model.scaffold23042.31	CREB1
Evm.model.scaffold23004.9	CNG3
Evm.model.scaffold17157.6	CLTA
Evm.model.scaffold18237.4	CHSY3
Evm.model.scaffold23000.8	CHRNB3
Evm.model.scaffold20628.4	CHRNA7
Evm.model.scaffold23000.7	CHRNA6
Evm.model.scaffold22671.19	CHKA
Evm.model.scaffold22717.2	CDKN1B
Evm.model.scaffold18267.7	CD274
Evm.model.scaffold21898.6	CCKAR
Evm.model.scaffold15230.13	CAMK2G
Evm.model.scaffold21641.8	BHMT
Evm.model.scaffold19995.7	B4GALT1
Evm.model.scaffold14602.10	ATP6V1G3
Evm.model.scaffold21641.6	ARSB
Evm.model.scaffold9906.14	AP4S1
Evm.model.scaffold15230.10	AP3M1
Evm.model.scaffold15230.53	ALOX5
Evm.model.scaffold21284.53	AGL
Evm.model.scaffold13692.5	ACTN1
Evm.model.scaffold12228.22	ACP1
Evm.model.scaffold22875.10	UNKNOWN
Evm.model.scaffold15230.49	ACOD
Evm.model.scaffold22860.1	ACO1

Evm.model.scaffold19805.6	ACER2
Evm.model.scaffold21754.59	ACADS
Evm.model.scaffold14488.20	ABI2
Evm.model.scaffold15230.51	ABCG2
Evm.model.scaffold21284.41	ABCD3
Evm.model.scaffold23016.24	ABCB11
Evm.model.scaffold21284.39	ABCA4
Evm.model.scaffold22850.4	ABCA1
Evm.model.scaffold22875.12	UNKNOWN
Evm.model.scaffold15230.9	UNKNOWN
Evm.model.scaffold3693.15	UNKNOWN
Evm.model.scaffold19995.1	UNKNOWN
Evm.model.scaffold12001.1	UNKNOWN
Evm.model.scaffold20994.9	UNKNOWN
Evm.model.scaffold23077.2	UNKNOWN
Evm.model.scaffold15230.24	UNKNOWN

Supplementary Table S3. Functional gene categories enriched for the high altitude (the eastern Himalayas) great tits compared to lowland (Central/East China and Mongolia) great tits. Only top 10 % categories are given below.

GO ID	Description	Number of genes	P-value
Biological process			
GO:0071840	Cellular component organization or biogenesis	23	6.474 E-03
GO:0007596	Blood coagulation	3	6.528 E-03
GO: 0007599	Hemostasis	3	6.528 E-03
GO:0050878	Regulation of body fluid levels	3	6.528 E-03
GO:0016043	Cellular component organization	21	7.088 E-03
GO:0050817	Coagulation	3	8.922 E-03
GO:0042060	Wound healing	3	1.062 E-02
GO:0051276	Chromosome organization	8	1.164 E-02
GO:0016568	Chromatin modification	4	1.312 E-02
GO:0071704	Organic substance metabolic process	176	1.538 E-02
GO:0043170	Macromolecule metabolic process	137	1.728 E-02
GO:0006325	Chromatin organization	7	1.738 E-02
GO:0008152	Metabolic process	202	2.135 E-02
GO:0044260	Cellular macromolecule metabolic process	120	2.153 E-02
GO:0044238	Primary metabolic process	171	2.176 E-02
Molecular function			
GO:0019887	Protein kinase regulator activity	4	6.216E-03
GO:0019207	Kinase regulator activity	4	1.228E-02
GO:0016705	Oxidoreductase activity	10	1.257E-02
GO:0004835	Tubulin-tyrosine ligase activity	3	1.4E-02
GO:0004889	Acetylcholine-activated cation-selective channel activity	3	2.0E-02
GO:0051213	Dioxygenase acitivty	5	2.194E-02
GO:0004725	Protein tyrosine phosphatase activity	6	2.43E-02
GO:0031072	Heat shock protein binding	6	2.80E-02 2.79E-02

Supplementary Table S4. Hypoxia genes showed signatures of selection in high altitude great tit. These genes exhibited a higher F_{ST}, θπ ratio and Tajima's *D* (Tajima's *D* lowland – Tajima's *D* highland) compared with the genome background.

Genes	F_{ST}	*P* value	Θπ ratio	*P* value	Tajima's *D* (lowland-highland)	*P* value
EP300	0.487	1.23E-29	3.452	3.16E-23	1.882	4.63E-21
PIK3CG	0.538	2.41E-25	2.820	1.77E-11	1.778	2.39E-14
CAMK2G	0.523	6.78E-59	3.373	7.73E-09	1.088	1.23E-11
MARK1	0.407	3.76E-15	2.210	6.90E-08	1.164	3.15E-09
NFKB2	0.43	9.30E-10	2.743	3.55E-04	0.914	1.04E-03
RPS6KA3	0.526	5.40E-25	2.8	1.24E-14	0.548	2.22E-05
PTPN5	0.556	2.15E-34	2.48	3.90E-10	1.228	6.95E-13
RPS6KA5	0.575	2.93E-22	4.375	1.34E-12	1.386	2.00E-08
CREB1	0.600	4.79E-11	5.6	1.08E-04	2.561	4.47E-11
F2R	0.512	4.82E-10	2.924	2.75E-05	0.918	2.41E-25
LAMB4	0.379	4.68E-23	2.078	1.77E-19	1.335	2.99E-12
LAMB1	0.466	2.10E-11	2.48	1.97E-07	0.467	5.01E-04
ITGA6	0.426	3.28E-25	2.496	8.03E-06	1.229	6.03E-06
ITGA2	0.463	3.65E-46	2.47	5.50E-10	0.523	2.47E-07
PRKAA1	0.337	1.84E-10	1.764	1.35E-03	0.546	4.71E-08
THBS4	0.624	3.66E-31	2.158	5.46E-06	0.951	1.56E-16
VWF	0.509	8.40E-62	2.017	3.60E-12	1.253	2.59E-28
CHRNA7	0.469	2.67E-25	2.179	4.06E-06	1.524	1.28E-21
ITPR2	0.459	3.52E-99	1.664	6.27E-11	0.777	8.61E-29
ALOX5	0.425	2.71E-20	1.869	8.35E-07	1.004	4.15E-07

Supplementary Table S5. Biometrical measurements of three groups of great tit.

	Body mass (BM)	Body length (BL)	Wing length (WL)	Tail length (TL)	Tarsus length (TL)	Bill length (BL)
Mongolia	18.01±0.75	151.78±3.70	74.02±0.66	68.64±0.72	18.78±0.12	9.00±0.13
Eastern Himalayas	15.72±0.28	137.11±1.70	73.64±0.59	65.98±0.65	18.15±0.17	8.05±0.17
Central/East	13.79±0.18	127.16±1.54	67.81±0.50	61.43±0.65	16.84±0.12	7.74±0.10

Supplementary Table S6. Positive selection in fatty-acid metabolism pathways of ground tit (*Parus humilis*). The data in this table are original from Figure 4 in [5].

Fatty acid metabolism<br>KEGG map 01212	4 genes	Aldehyde dehydrogenase (NAD+);<br>acyl-CoA oxidase;<br>acetyl-CoA C-acetyltransferase;<br>long-chain acyl-CoA synthetase
Biosynthesis of unsaturated fatty acids KEGG map 01040	4 genes	Acyl-CoA oxidase;<br>stearoyl-CoA desaturase;<br>enoyl reductase;<br>3-hydroxy acyl-CoA dehydratase)
Ether lipid metabolism<br>KEGG map 00565	6 genes	1-acyl-sn-glycerol-3-phosphate acyltransferase;<br>alkylglycerophosphoethanolamine phosphodiesterase;<br>alkyldihydroxyacetonephosphate synthase;<br>phospholipase A2;<br>phosphatidate phosphatase;<br>1-alkyl-2-acetylglycerophosphocholine esterase
Glycerolipid metabolism<br>KEGG map 00561	6 genes	Aldehyde dehydrogenase (NAD+);<br>1-acyl-sn-glycerol-3-phosphate acyltransferase;<br>glycerol kinase; diacylglycerol kinase;<br>acylglycerol lipase, phosphatidate phosphatase
Glycerophospholipid metabolism<br>KEGG map 00564	10 genes	Glycerol-3-phosphate dehydrogenase;<br>phospholipase A2;<br>choline O-acetyltransferase;<br>phosphatidate phosphatase;<br>1-acyl-sn-glycerol-3-phosphate acyltransferase;<br>diacylglycerol kinase;<br>phosphatidate cytidylyltransferase;<br>CDP-diacylglycerol--glycerol-3-phosphate 3-phosphatidyltransferase<br>phospholipase A1;<br>phosphatidylserine synthase 1

Supplementary Table S7. Sampling information of the great tit individuals used in this study. MON, Mongolian group, EH, the eastern Himalayas group, CE, the Central/East China group.

Vouch number	Localities	Group	Longitude	Latitude	Altitude
3918	Yanbian, Sichuan	EH	101.35.57	26.28.23	2200m
4000	Yanbian, Sichuan	EH	98.43.81	24.58.51	2200m
5226	Gaoligong, Yunnan	EH	98.43.81	24.58.51	2031m
5236	Gaoligong, Yunnan	EH	98.43.81	24.58.51	2031m
5242	Gaoligong, Yunnan	EH	98.43.81	24.58.51	2031m
5332	Gaoligong, Yunnan	EH	98.43.81	24.58.51	2031m
5333	Gaoligong, Yunnan	EH	98.43.81	24.58.51	2031m
13924	Lijiang, Yunnan	EH	100.07	27.10	2300m
13938	Lijiang, Yunnan	EH	100.07	27.10	2300m
13995	Lijiang, Yunnan	EH	100.07	27.10	2300m
13996	Lijiang, Yunnan	EH	100.07	27.10	2300m
11555	Eerguna, Inner Mongolia	MON	120.33.02	51.58.25	750m
11556	Eerguna, Inner Mongolia	MON	120.33.02	51.58.25	750m
11557	Eerguna, Inner Mongolia	MON	120.33.02	51.58.25	750m
11564	Eerguna, Inner Mongolia	MON	120.33.02	51.58.25	750m
15078	Teshig, Mongolia	MON	102.75.76	49.74.17	480m
15081	Teshig, Mongolia	MON	102.75.76	49.74.17	480m
15100	Tushig, Mongolia	MON	105.00.11	50.30.74	480m
15110	Tushig, Mongolia	MON	105.00.11	50.30.74	480m
15111	Tushig, Mongolia	MON	105.00.11	50.30.74	480m
17252	Altai Region, Russia	MON	85.65.83	51.62.87	500m
8204	Shenlongjia, Hubei	CE	110.92	31.88	750m
8236	Shenlongjia, Hubei	CE	110.92	31.88	750m
10076	Jixi, Anhui	CE	115.54	30.20	240m
10635	Panjin, Liaoning	CE	121.27.38	40.39.12	186m
10636	Panjin, Liaoning	CE	121.27.38	40.39.12	186m
10637	Panjin, Liaoning	CE	121.27.38	40.39.12	186m
10732	Kuandian, Liaoning	CE	124.85.32	40.58.66	186m

10860	Kuandian, Liaoning	CE	124.85.32	40.58.66	186m
11929	Sunan, Gansu	CE	102.00.46	37.50.23	780m
11944	Sunan, Gansu	CE	102.00.46	37.50.23	780m
12003	Yongdeng, Gansu	CE	102.44.12	36.41.08	780m

Supplementary Table S8. Statistics of reads mapping and coverage. MON, Mongolian group, EH, the eastern Himalayas group, CE, the Central/East China group.

Groups	Vouch number	High-quality base (bp)	Mapping rate (%)	Depth (×)	Coverage at least 1 × (%)	Coverage at least 4 × (%)
EH	3918	5.60E+09	82.09	3.95	90.11	53.93
EH	4000	5.77E+09	82.46	4.1	90.39	56.16
EH	5226	6.31E+09	84.04	4.53	90.73	61.53
EH	5236	6.45E+09	83.80	4.59	91.23	61.25
EH	5242	6.79E+09	84.07	4.86	91.21	65.38
EH	5332	5.99E+09	83.87	4.29	90.66	57.49
EH	5333	5.79E+09	81.57	4.06	90.22	55.56
EH	13924	6.00E+09	82.18	3.89	86.70	50.38
EH	13938	5.70E+09	84.54	4.12	90.37	56.39
EH	13995	5.44E+09	81.88	3.83	89.29	50.74
EH	13996	5.19E+09	82.17	3.67	89.39	49.25
MON	11555	5.91E+09	82.38	4.15	90.57	56.08
MON	11556	6.68E+09	78.97	4.43	90.66	60.11
MON	11557	6.23E+09	81.48	4.29	90.48	58.71
MON	11564	5.22E+09	78.71	3.52	89.08	45.72
MON	15078	5.76E+09	82.02	3.99	90.01	54.42
MON	15081	6.64E+09	79.21	4.42	91.00	59.39
MON	15100	5.38E+09	82.95	4.17	90.43	57.24
MON	15110	5.21E+09	80.37	3.57	89.30	47.06
MON	15111	5.31E+09	79.12	3.57	89.27	46.48
MON	17252	6.00E+09	81.42	4.11	90.31	56.20
CE	8204	5.70E+09	82.05	3.96	89.55	52.51
CE	8236	5.59E+09	83.88	4.00	90.23	54.92
CE	10076	6.07E+09	83.38	4.31	90.52	58.91
CE	10635	6.15E+09	81.51	4.25	90.63	58.08
CE	10636	5.47E+09	83.02	3.85	89.86	52.20
CE	10637	5.96E+09	79.97	4.04	90.00	53.96
CE	10732	5.00E+09	80.01	3.45	88.57	44.81
CE	10860	6.00E+09	82.18	3.89	86.70	50.38
CE	11929	5.95E+09	82.37	4.15	90.63	56.07
CE	11944	5.93E+09	82.95	4.17	90.43	57.24
CE	12003	6.00E+09	83.49	4.05	90.44	54.30

Supplementary Table S9. Statistics of SNPs in whole genome of great tits.

	Number of SNPs	Θπ (10^{-3})	ΘW(10^{-3})	Non-synonymous SNPs	Synonymous SNPs	Nonsy/Syn
Mongolia	2,237,921	3.147	2.509	9,313	35,941	0.2591
Eastern Himalayas	2,999,524	5.185	3.971	11,728	46,932	0.2499
Central/East China	3,093,290	5.302	4.145	12,118	48,375	0.2505

Figure S1

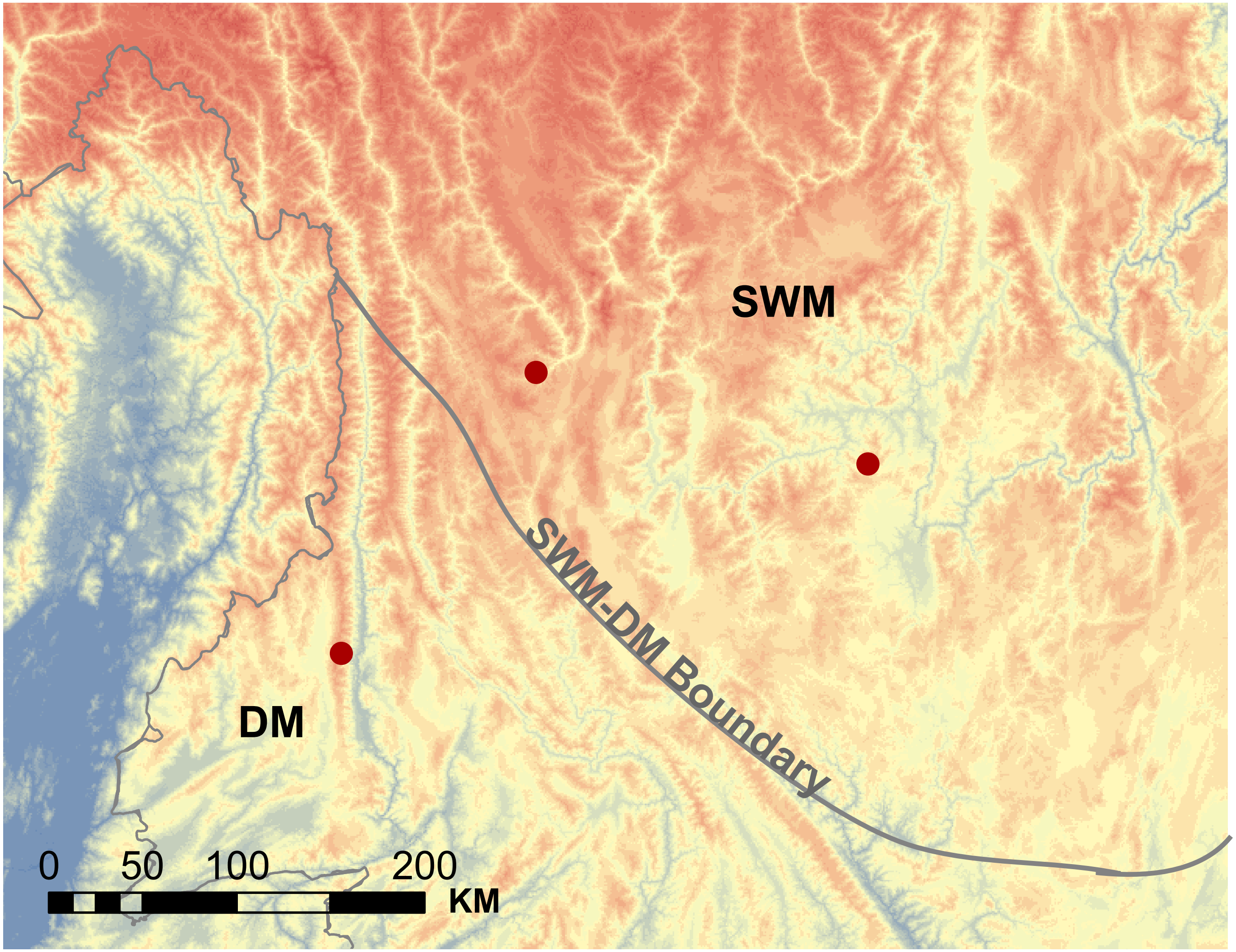

Figure S2

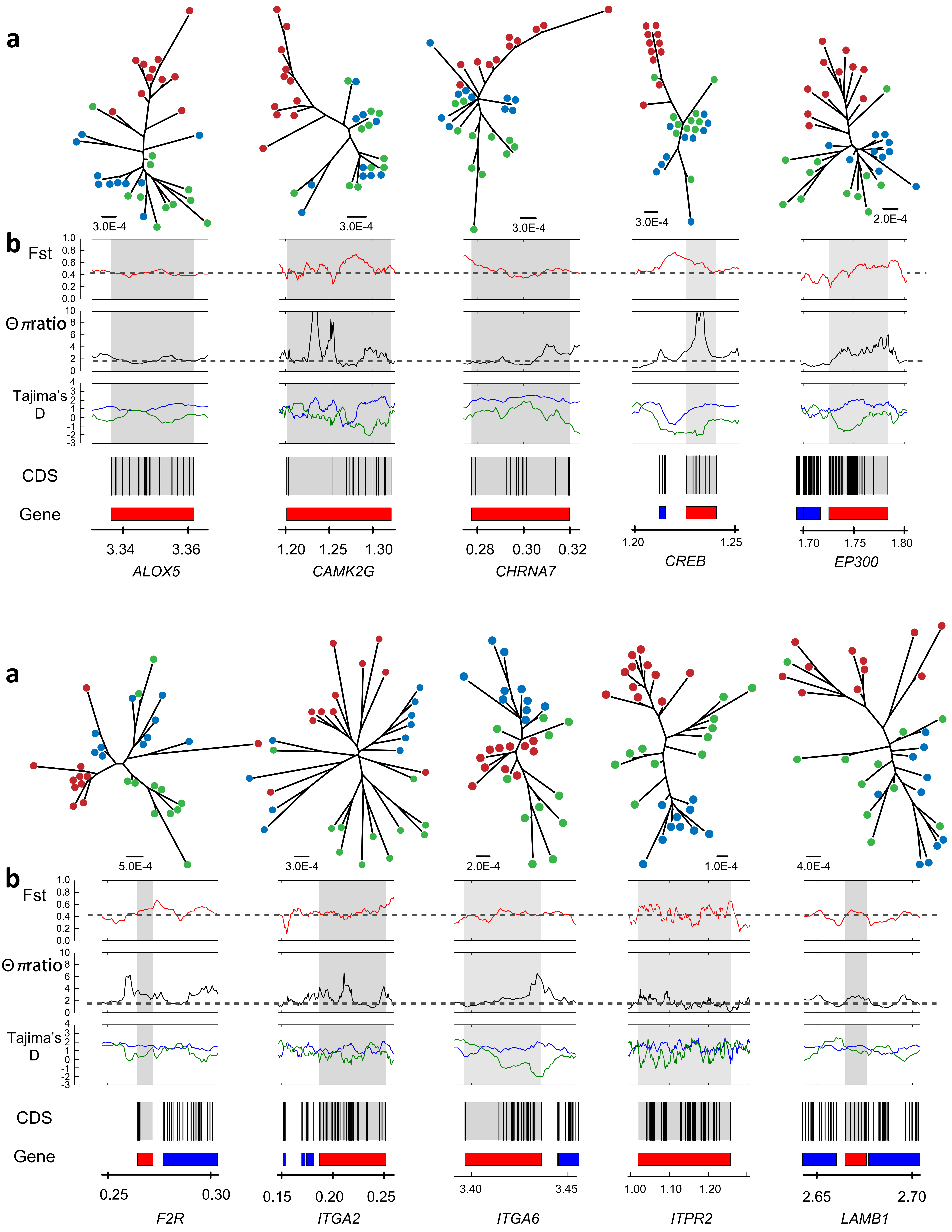

a
b
Fst
Θπratio
Tajima's D
CDS
Gene
3.0E-4
2.0E-4
ALOX5
CAMK2G
CHRNA7
CREB
EP300
F2R
ITGA2
ITGA6
ITPR2
LAMB1
5.0E-4
1.0E-4
4.0E-4

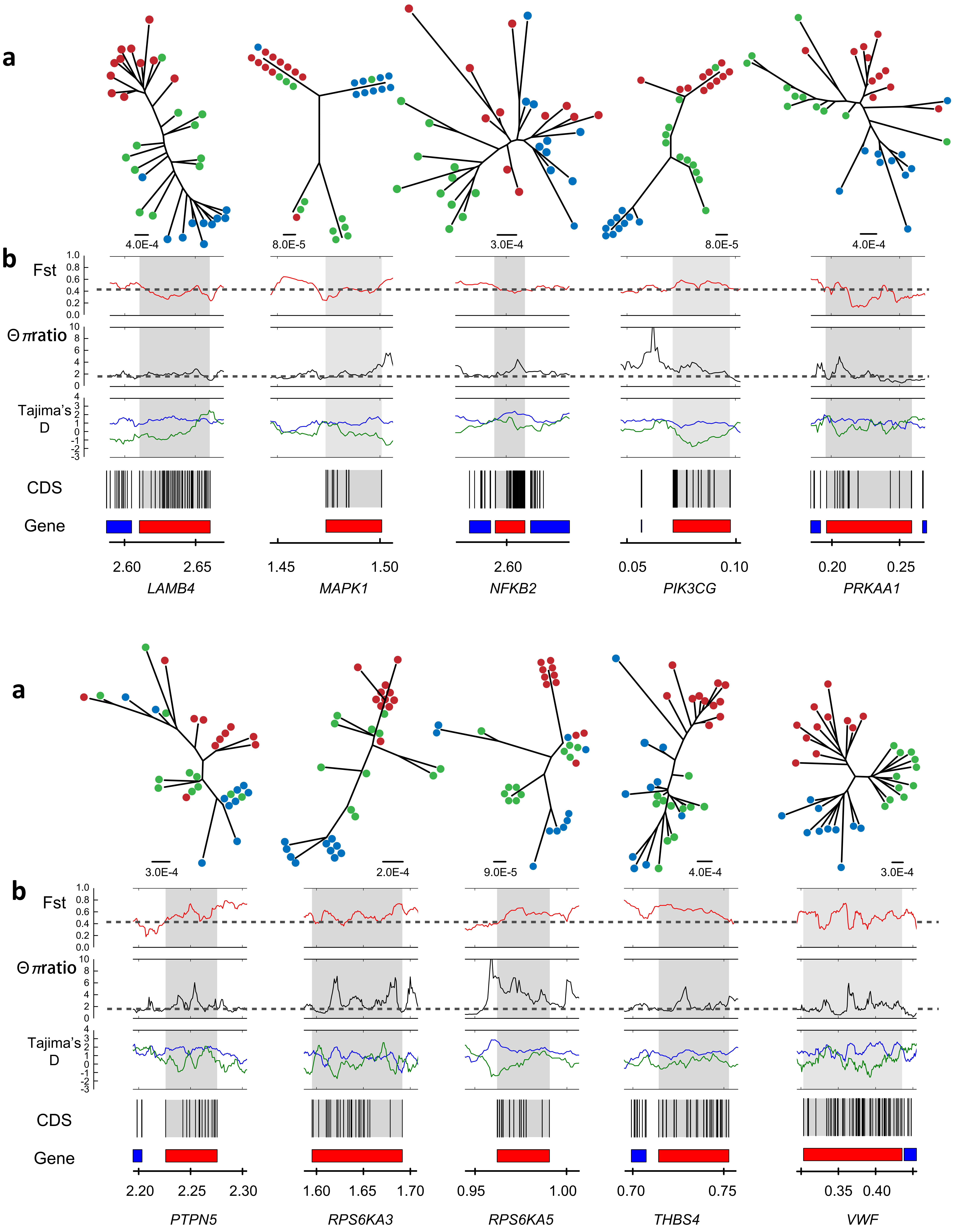
a
4.0E-4
8.0E-5
3.0E-4
8.0E-5
4.0E-4
b
Fst
Θπratio
Tajima's D
CDS
Gene
2.60
2.65
1.45
1.50
2.60
0.05
0.10
0.20
0.25
LAMB4
MAPK1
NFKB2
PIK3CG
PRKAA1
a
3.0E-4
2.0E-4
9.0E-5
4.0E-4
3.0E-4
b
Fst
Θπratio
Tajima's D
CDS
Gene
2.20
2.25
2.30
1.60
1.65
1.70
0.95
1.00
0.70
0.75
0.35
0.40
PTPN5
RPS6KA3
RPS6KA5
THBS4
VWF

Figure S3

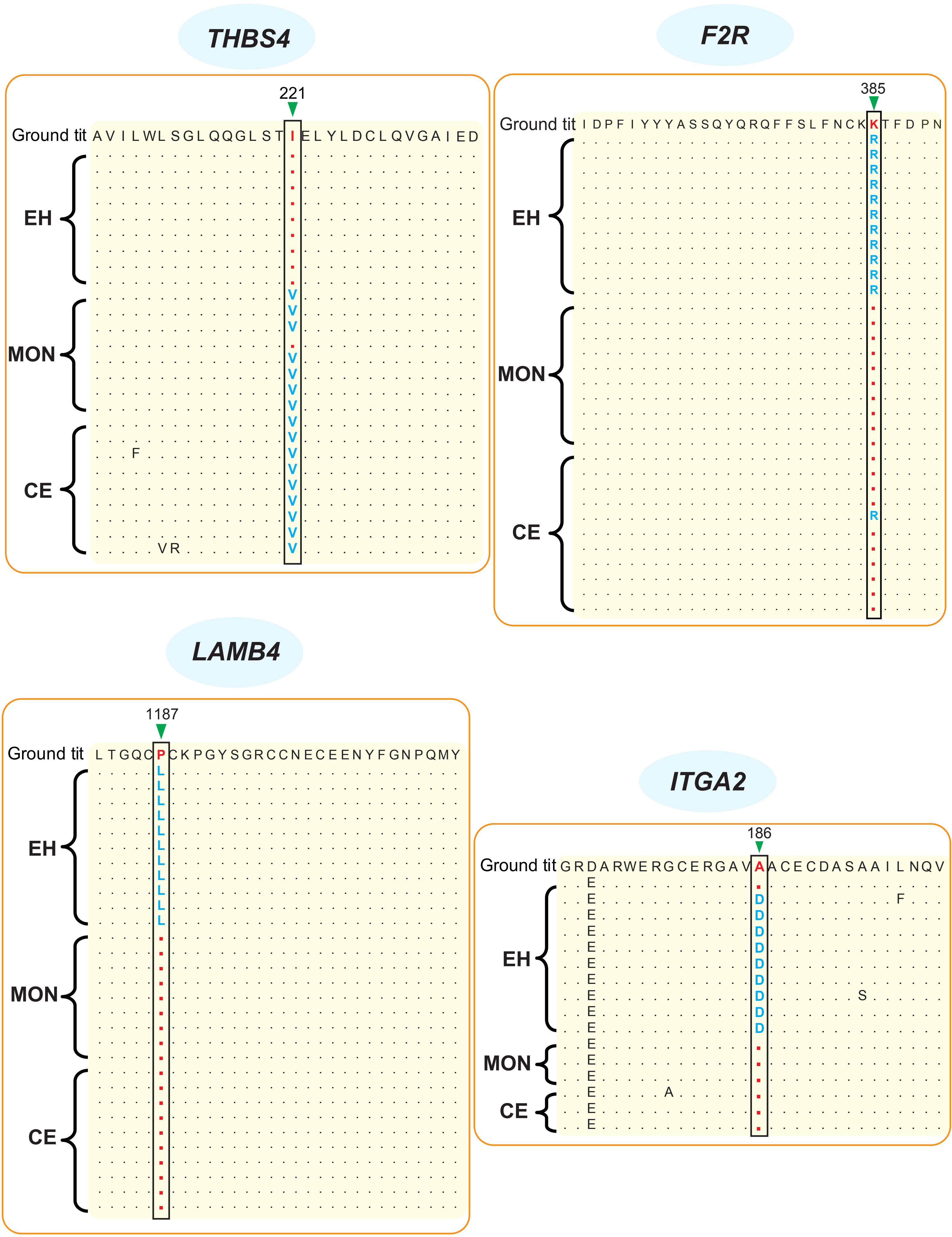
THBS4
221
Ground tit A V I L W L S G L Q Q G L S T I E L Y L D C L Q V G A I E D
EH
MON
CE
F2R
385
Ground tit I D P F I Y Y Y A S S Q Y Q R Q F F S L F N C K K T F D P N
EH
MON
CE
LAMB4
1187
Ground tit L T G Q C P C K P G Y S G R C C N E C E E N Y F G N P Q M Y
EH
MON
CE
ITGA2
186
Ground tit G R D A R W E R G C E R G A V A A C E C D A S A A I L N Q V
EH
MON
CE

Figure S4

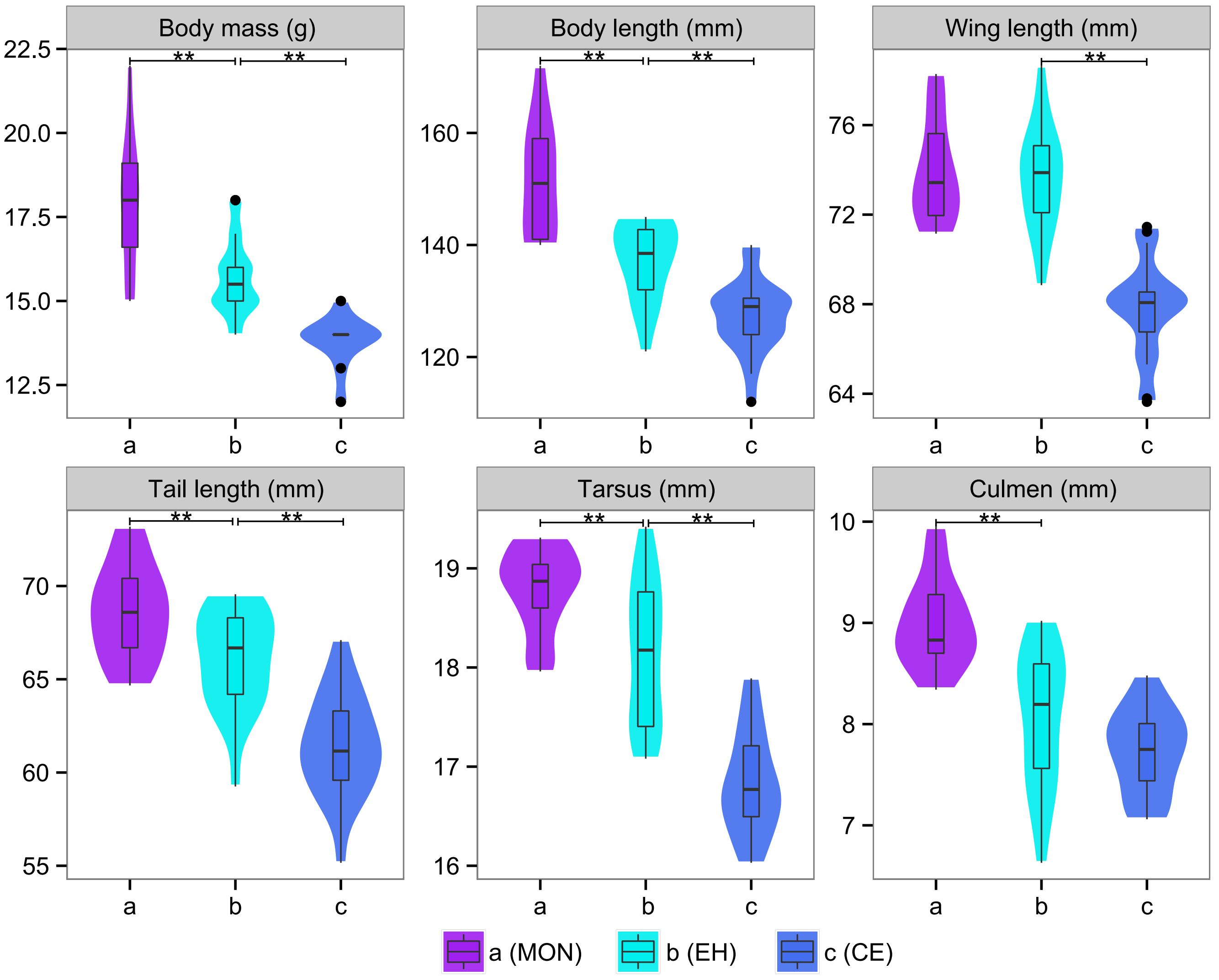

Body mass (g)
Body length (mm)
Wing length (mm)
Tail length (mm)
Tarsus (mm)
Culmen (mm)
22.5
20.0
17.5
15.0
12.5
160
140
120
76
72
68
64
70
65
60
55
19
18
17
16
10
9
8
7
**
a
b
c
a (MON)
b (EH)
c (CE)
